# Fine-scale genetic breaks driven by historical range dynamics and ongoing density-barrier effects in the estuarine seaweed *Fucus ceranoides* L.

**DOI:** 10.1186/1471-2148-12-78

**Published:** 2012-06-06

**Authors:** João Neiva, Gareth A Pearson, Myriam Valero, Ester A Serrão

**Affiliations:** 1 Centro de Ciências do Mar, Centro de Investigação Marinha e Ambiental - Laboratório Associado, Universidade do Algarve, Gambelas, Faro, 8005-139, Portugal; 2 Unité Mixte de Recherche 7144, Centre National de la Recherche Scientifique/Université Pierre et Marie Curie, Station Biologique de Roscoff, Place Georges-Teissier, BP 74, Roscoff Cedex, 29682, France

## Abstract

**Background:**

Factors promoting the emergence of sharp phylogeographic breaks include restricted dispersal, habitat discontinuity, physical barriers, disruptive selection, mating incompatibility, genetic surfing and secondary contact. Disentangling the role of each in any particular system can be difficult, especially when species are evenly distributed across transition zones and dispersal barriers are not evident. The estuarine seaweed *Fucus ceranoides* provides a good example of highly differentiated populations along its most persistent distributional range at the present rear edge of the species distribution, in NW Iberia. Intrinsic dispersal restrictions are obvious in this species, but have not prevented *F. ceranoides* from vastly expanding its range northwards following the last glaciation, implying that additional factors are responsible for the lack of connectivity between neighbouring southern populations. In this study we analyze 22 consecutive populations of *F. ceranoides* along NW Iberia to investigate the processes generating and maintaining the observed high levels of regional genetic divergence.

**Results:**

Variation at seven microsatellite loci and at mtDNA spacer sequences was concordant in revealing that Iberian *F. ceranoides* is composed of three divergent genetic clusters displaying nearly disjunct geographical distributions. Structure and AFC analyses detected two populations with an admixed nuclear background. Haplotypic diversity was high in the W sector and very low in the N sector. Within each genetic cluster, population structure was also pervasive, although shallower.

**Conclusions:**

The deep divergence between sectors coupled with the lack of support for a role of oceanographic barriers in defining the location of breaks suggested 1) that the parapatric genetic sectors result from the regional reassembly of formerly vicariant sub-populations, and 2) that the genetic discontinuities at secondary contact zones (and elsewhere) are maintained despite normal migration rates. We conclude that colonization and immigration, as sources of gene-flow, have very different genetic effects. Migration between established populations is effectively too low to prevent their differentiation by drift or to smooth historical differences inherited from the colonization process. *F. ceranoides*, but possibly low-dispersal species in general, appear to be unified to a large extent by historical, non-equilibrium processes of extinction and colonization, rather than by contemporary patterns of gene flow.

## Background

Marine ecosystems have historically been considered to be relatively open, with populations demographically and genetically connected over broad spatial scales. In a range of coastal taxa, however, recent molecular surveys have consistently revealed considerable phylogeographical and population genetic structure, often at seemingly small spatial scales, indicating that connectivity is frequently much lower than previously assumed. Examples include a variety of species lacking planktonic dispersive stages and/or exhibiting particularly strict ecological requirements, such as intertidal fucalean and kelp seaweeds [1-5], seagrasses [6,7], direct-developing invertebrates and fish [8-11], high-intertidal rock-pool invertebrates [12], and many estuarine organisms [13-18]. In such species, shallow genetic discontinuities can be common due to intrinsic life-history and habitat constraints to dispersal. Often, however, they also display deeper genealogical splits that distinguish regional sets of populations across their ranges. Such nested patterns of phylogeographical structure can result from a number of factors and are frequently harder to interpret, especially when species are evenly distributed across transition zones.

Vicariance is usually invoked as the main driver of (neutral) genetic divergence. Extrinsic barriers to gene-flow are generally less obvious (or absolute) in marine compared to terrestrial landscapes [19,20], but circulation patterns, coastline topography and habitat discontinuities have all been shown to potentially represent effective barriers to the exchange of individuals between adjacent marine regions [21-24]. Complex variations in habitat availability and connectivity, resulting from the Pleistocene oscillations in sea levels and surface temperatures, are also known to have produced ancient population subdivisions (and differentiation) in many coastal organisms [6,25,26]. Within a species, disjunct distribution of divergent genetic lineages provides strong indication for the occurrence of such vicariant processes.

Inferring the existence of a particular dispersal barrier from molecular data may not be straightforward though [27]. In species with short dispersal range, discontinuities in individual gene trees (mostly derived from organelle markers) readily arise haphazardly within continuously distributed species simply as a consequence of idiosyncratic lineage sorting [28,29]. Similarly, genetic drift during spatial expansions [30] or disruptive selection [31] can also result in the geographic segregation of organelle lineages across a species range even in the face of dispersal. In general, long-term isolation can only be confidently assumed when spatially concordant patterns across multiple unlinked loci are found [29,32].

Disentangling historical from ongoing constraints to dispersal may also be problematic. Phylogeographical breaks and contemporary oceanographic barriers (or biogeographical transition zones) are often mismatched in marine restricted dispersers [33]. Historical patterns of isolation and colonization in these organisms explain population structure better than more recent factors affecting gene-flow. Phylogeographical breaks may develop where formerly vicariant sub-populations have reassembled. The Iberian peninsula is a good example where diverse taxa such as trees [34], amphibians [35,36], reptiles [37,38] and pond-dwelling invertebrates [39,40] are sub-divided into well defined, mostly parapatric genetic sectors that presumably formed during expansions from disjunct refugia. The temporal persistence of genetic discontinuities across marine secondary contact zones have also been demonstrated in several species [26]. However, insight into the processes preventing steady genetic homogenization of divergent but contacting gene-pools requires finer scale genetic sampling than is common in most studies (but see [3,6,9,12,41,42]).

Virtually all coastal organisms have some potential to disperse and colonize new habitats, as the extensive post-glacial range shifts of many demonstrate. Thus, migration would also be expected to occur between fully established populations, including between divergent populations in relatively close proximity. Incipient reproductive isolation can reduce or prevent gene flow between divergent contacting lineages [41,43]. The persistence of fine-scale genetic differentiation in the absence of obvious reproductive and dispersal barriers seems paradoxical. In restricted dispersers, however, colonization and immigration, as sources of gene-flow, may have very different genetic effects. During expansions into vacant habitats, the original colonists can grow exponentially and contribute disproportionately to the genetic composition of the establishing population. In contrast, once the habitat patch is filled, demographic stability and increased competition can considerably reduce the impact of subsequent immigrants [44,45]. In addition, if there is a gross disparity between the number of residents and immigrants, a common situation in low dispersal species, foreign genotypes introduced in a population will *a priori* be rare and have low probability of random increase due to drift alone [46-48]. In other words, established populations themselves can create a density-barrier effect buffering local changes in allele frequencies and delaying the spatial advance of genes within previously colonized areas (despite immigration). At broad geographical scales, such an effect has been invoked to explain the persistence of genetic homogeneity in recolonized areas [44], the asymmetrical introgression of genes from established to spatially expanding species [49], or the lack of gene-flow between former refugial areas that are currently connected by intermediate populations [50]. When effective migration rates are low, patterns of non-equilibrium divergence resulting from founder and density-barrier effects can occur at much smaller spatial scales [46,51].

In this study, we report a remarkable case of non-equilibrium divergence in the estuarine seaweed *Fucus ceranoides*, in which steep genetic discontinuities are preserved despite the absence of obvious barriers to dispersal. *Fucus ceranoides* L. (horned wrack) is a perennial, dioecious seaweed restricted to estuarine environments across much of the Northeast Atlantic. Populations of *F. ceranoides* from NW Iberia, at the rear edge of the species distribution, form three highly divergent genetic clusters according to both mtDNA and microsatellite markers [15,52]. Despite their relatively close proximity (~150 km), fixed genetic differences at this scale suggest that the historical and recurrent processes contributing to their differentiation are weakly counteracted by on-going gene-flow. The poor dispersal ability of *F. ceranoides* certainly plays a role; fucoid algae lack planktonic dispersive stages and therefore *F. ceranoides* individuals typically complete their entire life-cycle within the discrete, isolated patches of the estuarine habitat they inhabit. Still, an important question remains unanswered concerning the nature and stability of genetic divergence in this system. Like in many other seaweeds, non-local (inter-estuarine) dispersal can be mediated by rafting of detached, reproductive individuals [53,54]. Such dispersal by drifting thalli was likely responsible for the extensive post-glacial expansion of *F. ceranoides* into Northern Europe, including the distant colonization of Norway (across the North Sea) and Iceland [15,52]. If *F. ceranoides* managed to expand its range more than 15 degrees in latitude since the Last Glacial Maximum [LGM, ~20.000 ka before present (BP)], dispersal restrictions cannot account, at least as the sole factor, for the apparent lack of population connectivity along the much narrower NW Iberian coastline.

This study aims to understand this fundamental issue in the evolutionary ecology of populations, the apparently contradicting evidence for large scale dispersal mediating vast (re)colonisations concurrently with persistent, fine scale genetic discontinuities in older refugial regions. The specific question is whether such discontinuities arise and persist due to long-lasting dispersal barriers, or simply reflect resilient non-equilibrium conditions inherited from a complex demographic past. To address this question, in this study both mtDNA sequence and microsatellite genotypic data are employed to investigate the fine-scale distribution of genetic variation in *F. ceranoides* from NW Iberia. This region was sampled at the finest scale of resolution achievable—a complete set of neighbouring estuaries—which was the scale over which gene-flow was more likely to be detected. We were particularly interested in the biogeographic context and the demographic processes contributing to the formation and integrity of stable genetic sectors in NW Iberian *F. ceranoides*.

## Results

### MtIGS phylogeography

**Figure 1:**
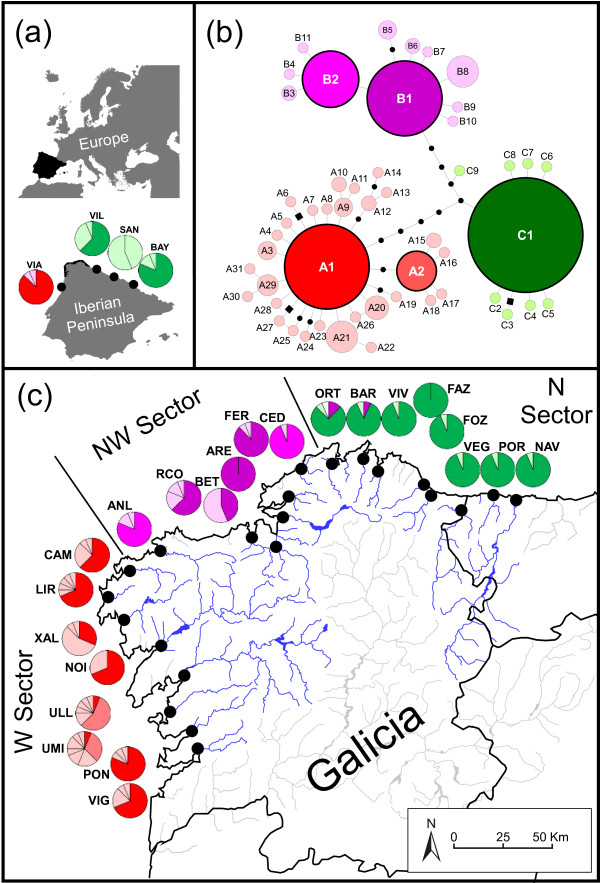
**Genealogy and distribution of the mtIGS haplotypes of *Fucus ceranoides *from NW Iberia.** (**a**) Location of the study area (in black) in relation to Europe and the Iberian Peninsula. The geographical location and mtIGS lineages present in four Iberian populations previously analysed in Neiva *et al.* (2010, 2012) are also shown. (**b**) MtIGS parsimony networks of NW Iberian haplotypes. Sampled haplotypes are represented by circles sized to their frequency and black dots represent inferred, unsampled haplotypes. Links represent a single nucleotide change and black squares represent small indels. Inferred phylogroups are labelled by colour and letter (A-Red; B-Purple; C-Green). Shared and private haplotypes are depicted in bright and pale colour intensity, respectively. (**c**) Location of sampling sites, delimitation of the phylogeographic sectors considered (Western- W; Northwestern- NW; Northern- N). Pie charts depict haplotype frequencies at each site (see Table 1 for haplotype ID’s, haplotypes are coloured as in b)

**Figure 2:**
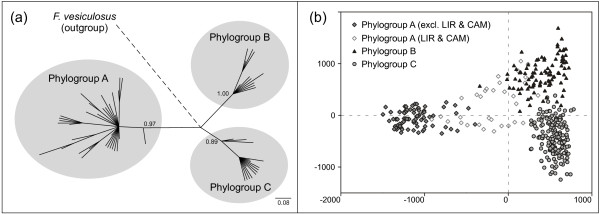
**MtIGS phylogeny and genealogic congruence in Iberian *Fucus ceranoides. *** (**a**) 50% majority-rule consensus tree of mtIGS haplotypes of *F. ceranoides*, rooted with *F. vesiculosus*. Numbers above the branches are Bayesian posterior probabilities (> 0.70). Inferred phylogroups are highlighted in grey. (**b**) FCA plot based on individual multilocus genotypes. Individuals are labelled according to their mtIGS lineage. Note the correspondence between the mtIGS phylogeny and the nuclear population structure. The individuals from VIA, VIL, SAN and BAY were included in both analyses.

A total of 51 mtIGS haplotypes (GenBank: JN084346-96) were identified in the 352 individuals of *F. ceranoides* belonging to the 22 “core” populations. The median-joining network revealed three mtIGS lineages displaying nearly disjunct geographic distributions (Table 1 and Figure 1). The Bayesian phylogenetic analyses failed to recover the temporal sequence of lineage splitting, but their monophyly was supported by high branch posterior probabilities (Figure 2a). Each phylogroup was defined by one interior and widespread haplotype. Phylogroup A, composed by haplotypes *A1* and 30 related ones, was present from VIG to CAM (Western sector, W), and further south in VIA. Phylogroup B, composed by *B1* and 10 related haplotypes, was distributed from ANL to CED (North-Western sector, NW), although a few B haplotypes were also detected in ORT and BAR. Finally, phylogroup C, composed by *C1* and 8 related haplotypes, was exclusively found from ORT eastwards to NAV (Northern sector, N), and further east in the populations of VIL, SAN and BAY. Several peripheral populations were geographically closer to populations across the phylogeographic breaks than they were to their nearest population within the same sector. For instance, ANL (NW sector) is geographically closer to CAM (W sector; ~34 km) than to RCO (NW sector, ~70 km), and the distance between CED (NW sector) and ORT (N sector; ~36,5 km) is smaller than between CED and FER (~58 km; both NW sector).

**Table 1:** **Genetic diversity of *Fucus ceranoides *within sampling sites and inferred genetic sectors**

**River (Ria), Village**	**Code**	**N**	**Microsatellites**				**MtIGS**				
			**A**	**H**_**E**_	**H**_**O**_	**F**_**IS**_	**Lineage**	**Haplotypes**	**N**_**hap**_	**H**_**hap**_**(10**^**−3**^**)**	**Π**_**hap**_**(10**^**−5**^**)**
Lima, Viana do Castelo	VIA	16	2.43	0.154	0.143	0.077	A	A1(14), GQ385159*, GQ385159*	3	242	50
**Western Sector**	**W**	**128**	**9.00**	**0.457**	**0.257**		**A**	**A1–A31**	**31**	**717**	**351**
Verdugo (Ria de Vigo), Arcade	VIG	16	3.57	0.223	0.223	−0.001	A	A1(11), A3(3), A4, A5	4	517	116
Lérez (Ria de Pontevedra), Pontevedra	PON	16	2.29	0.174	0.161	0.080	A	A1(13), A6, A7, A8	4	242	50
Umia (Ria de Arousa), Cambados	UMI	16	2,86	0.225	0.174	0.231*	A	A1, A2(5), A9(3), A10 (2), A11, A12(2), A13, A14	8	875	580
Ulla (Ria de Arousa), Catoira	ULL	16	2,71	0.144	0.146	−0.012	A	A1, A2(9), A15(2), A16, A17, A18, A19	7	692	267
Tabra/Tambre (Ria de Muros e Noia), Noia	NOI	16	3.00	0.357	0.370	−0.039	A	A1(11), A20(5)	2	458	92
Xallas (Ria de Córcubion), Ézaro	XAL	16	3.71	0.478	0.369	0.234*	A	A1 (5), A21(9), A22, A23	4	617	151
Castro, Lires	LIR	16	3.29	0.447	0.324	0.282*	A	A1 (11), A24, A25, A26, A27, A28	6	350	104
Grande (Ria das Camariñas), Ponte do Porto	CAM	16	3.43	0.430	0.279	0.360*	A	A1(10), A29(4), A30, A31	4	575	151
**North-Western Sector**	**NW**	**96**	**6.57**	**0.526**	**0.327**		**B**	**B1–B11**	**11**	**671**	**186**
Anllóns (Ria de Corme e Laxe), Ponteceso	ANL	16	3.57	0.482	0.482	0.001	B	B2 (13), B3(2), B4	3	342	72
Mero (Ria de A Coruña), O Temple	RCO	16	4.00	0.355	0.304	0.150*	B	B1 (10), B5(3), B6(2), B7	4	592	203
Mendo/Mandeo (Ria de Betanzos), Betanzos	BET	16	3.14	0.364	0.265	0.277*	B	B1(7), B8 (9)	2	525	106
Eume (Ria de Ares), Pontedeume	ARE	16	4.00	0.408	0.342	0.166*	B	B1(14), B9, B10	3	242	50
Xuvia (Ria do Ferrol), Neda	FER	16	3.71	0.458	0.368	0.203*	B	B1(16)	1	–	–
Ferrerias (Ria de Cedeira)	CED	16	3.00	0.398	0.200	0.505*	B	B2(15), B11	2	125	25
**Northern Sector**	**N**	**128**	**6.29**	**0.553**	**0.441**		**B,C**	**B1, C1–C9**	**10**	**166**	**144**
Mera (Ria de Ortigueira), Ponte de Mera	ORT	16	3.00	0.468	0.362	0.232*	B, C	B1(2), C1(12), C2, C9	4	442	323
Sor (Ria de Barquero), Poceira	BAR	16	3.14	0.563	0.411	0.277*	B, C	B1, C1(14), C3	3	133	113
Landro (Ria de Viveiros), Viveiros	VIV	16	3.57	0.484	0.473	0.022	C	C1(15), C4	2	125	25
Ouro	FAZ	16	3.43	0.497	0.500	−0.007	C	C1(15), C5	2	125	25
Masma (Ria da Foz)	FOZ	16	3.57	0.507	0.427	0.162*	C	C1(16)	1	–	–
Eo (Ria de Ribadeo), Vegadeo	VEG	16	3.71	0.416	0.414	0.005	C	C1(15), C6	2	125	25
Porcia	POR	16	3.00	0.463	0.527	−0.144	C	C1(15), C7	2	125	25
Navia (Ria de Navia), Navia	NAV	16	3.71	0.464	0.413	0.114	C	C1(15), C8	2	125	25
Valdediós (Ria de Villaviciosa)	VIL	16	3.00	0.464	0.464	0.000	C	C1(10), GQ385159*(5), GQ385159*	3	542	117
Asón (Ria de Santoña), Colindres	SAN	16	3.14	0.410	0.411	−0.003	C	GQ385159*(9), GQ385159*(7)	2	525	105
Adour, Bayonne	BAY	16	3.43	0.486	0.446	0.083	C	C1(13), GQ385159*(2), GQ385159*	3	342	72

Mean allelic richness (A), Nei’s gene diversity (*H*_E_), observed heterozygosity (*H*_o_) and multi-locus inbreeding coefficient (*F*_IS_,) were estimated from for the microsatellite data-set; Haplotypic richness (*N*_hap_), haplotypic diversity (*H*_hap_) and nucleotide diversity (*π*_hap_) are based on the mtIGS data-set. The mtIGS lineages and haplotypes are listed for each population (coded as in Figure 1). Absolute frequencies of haplotypes are in parenthesis (if N > 1). *GenBank accessions of the private haplotypes of the populations of VIA, VIL, SAN and BAY [15].

Globally, only the three dominant haplotypes (*A1*, *B1* and *C1*) plus two derived ones (*A2*, *B2*) were shared among at least two populations. The remaining 46 haplotypes were population-specific and among these, 12 represented non-singleton variants. Many W and NW populations harboured private haplotypes in relatively high frequencies. This pattern was apparent even in populations located inside the same drainage systems, such as UMI and ULL (Ria de Arousa, W sector), or RCO, BET, ARE and FER (Artabro Gulf, NW sector). *H*_hap_ was high in the W (*H*_hap_ = 0.717) and NW (*H*_hap_ = 0.671) sectors due to the presence of most of the local haplotype radiations, but *π*_hap_ was considerably higher in the former. Contrastingly, *H*_hap_ was very low in the N sector (*H*_hap_ = 0.166), but further east the populations of VIL and SAN possessed private, C1-derived haplotypes in relatively high frequencies.

**Figure 3:**
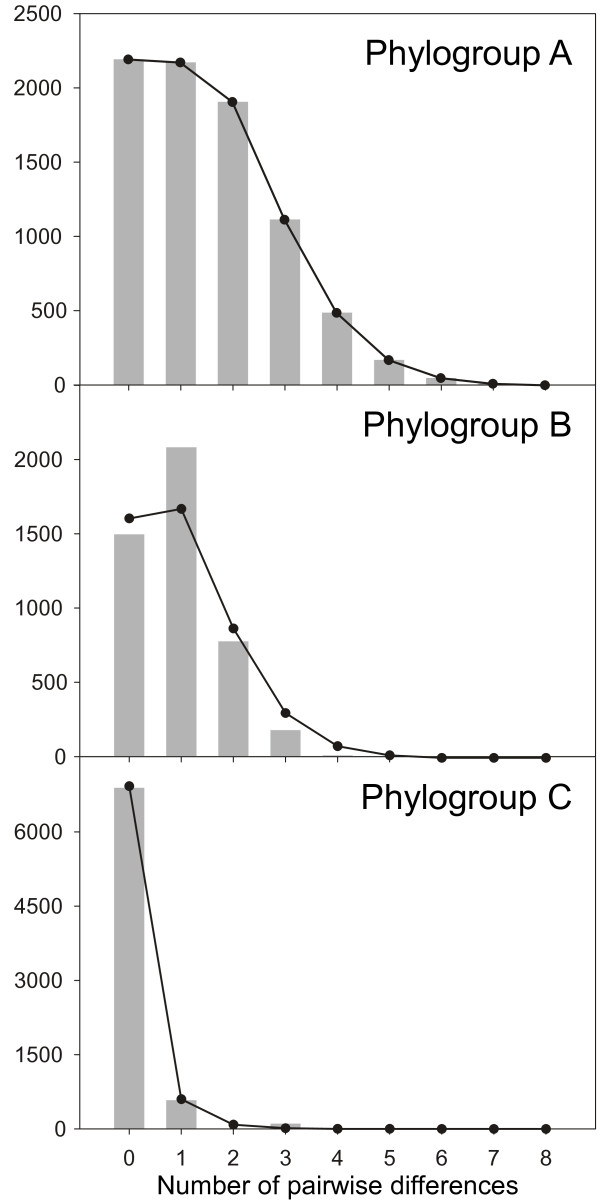
**Mismatch distributions of the mtIGS phylogroups A, B and C of *Fucus ceranoides. *** The grey bars and the solid lines depict the observed and expected (under the spatial expansion model) values, respectively.

The results of the AMOVAs showed that the sectors considered accounted for about 83% of the molecular variance of NW Iberian *F. ceranoides* (Table 2). Within the W, NW and N sectors, 32%, 63% and 2% of the molecular variance was accounted for by the molecular differences among respective populations. The mismatch distributions did not reject the spatial expansion of phylogroups A (*P* = 0.957) and C (*P* = 0.750), but failed to support the expansion of phylogroup B (*P* = 0.009; Figure 3).

**Table 2:** **Analyses of molecular variance (AMOVA) between and among NW Iberian genetic sectors of *F. ceranoides ***

***Analysis***	***N***	***Level***	***d.f.***	***Variance (%)***	***Fixation indices***
3 Sectors	352	Among groups	2	83.07	Φ_CT_ = 0.831*
		Among populations within groups	19	5.55	Φ_SC_ = 0.328*
		Among populations	330	11.38	
W Sector	128	Among populations	7	32.01	Φ_ST_ = 0.320*
		Within populations	120	67.99	
NW Sector	96	Among populations	5	63.44	Φ_ST_ = 0.634*
		Within populations	90	36.56	
N Sector	128	Among populations	7	2.24	Φ_ST_ = 0.023
		Within populations	120	97.76	

*P* values are based on 1000 permutations.

*P > 0.05.

### Microsatellite population structure

The seven microsatellite loci revealed a total of 76 alleles in the 352 “core” individuals genotyped (6–23 per locus), although 42% of these had global frequencies below 0.01. *H*_E_ was rather variable among populations, ranging from 0.144 (ULL; W sector) to 0.563 (BAR, N Sector) (Table 1). Approximately half of the populations exhibited significant heterozygote deficiencies. Among sectors, *H*_E_ and *H*_hap_ were not correlated (Additional file 1: Figure S1a and S1b). The W sector had the lowest (yet most variable) *H*_E_ (0.457) and the highest *H*_hap_ (0.717), whereas the N sector had the highest *H*_E_ (0.553) despite very low *H*_hap_ (0.166). The NW sector showed intermediate levels of diversity for both markers. FST ranged from 0.021 (VIG vs. UMI) to 0.685 (PON vs. CED), whereas *D*_est_ ranged from <0.001 (VIG vs. PON) to 0.879 (PON vs. ANL) (Additional file 2: Table S1). Within sectors, pairwise differentiation of populations was of the same order of magnitude, but more variable within the W sector (Additional file 1: Figure S1c).

**Figure 4:**
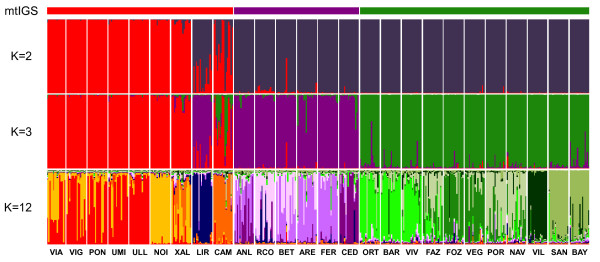
**Genetic subdivision of Iberian *Fucus ceranoides *based on STRUCTURE.** Shown are the proportions of individual multilocus genotypes assigned to each of K virtual clusters, as illustrated by the different colours. The individuals from VIA, VIL, SAN and BAY were also included. Population codes are given in Table 1.

The microsatellite genotypic clusters recovered with the FCA showed a remarkable correspondence with the mtIGS phylogroups (Figure 2b). The most obvious exceptions were the populations of LIR and CAM, both belonging to the W sector, whose genotypes appeared intermediate between W and NW and W and N sectors, respectively. Excluding these admixed populations, the W populations formed the most differentiated cluster among the three, as in the phylogenetic tree (Figure 2a). The STRUCTURE analyses showed a similar picture (Figure 4). Based on the ΔK ad-hoc criterion [55] the highest hierarchical level of genetic sub-division of *F. ceranoides* occurred between the W sector and the NW and N sectors (K = 2; Additional file 3: Figure S2). Further subdivision of genotypes into W, NW and N sectors (K = 3) represented a weaker, but nevertheless significant, level of population subdivision. Again, the individuals of LIR and CAM showed variable degree of admixture between the W and the NW and N sectors, respectively. Within these 3 major groups some sub-structuring was also evident (Figure 4). Iberian *F. ceranoides* could be subdivided into a maximum of 12 (stable) genetic clusters (K = 12), corresponding to smaller, less resolved geographic regions. Some admixture (or mixed-ancestry) between neighbouring clusters was pervasive, but also apparent in a few well separated population pairs (e.g. LIR & RCO, ANL & CED).

**Figure 5:**
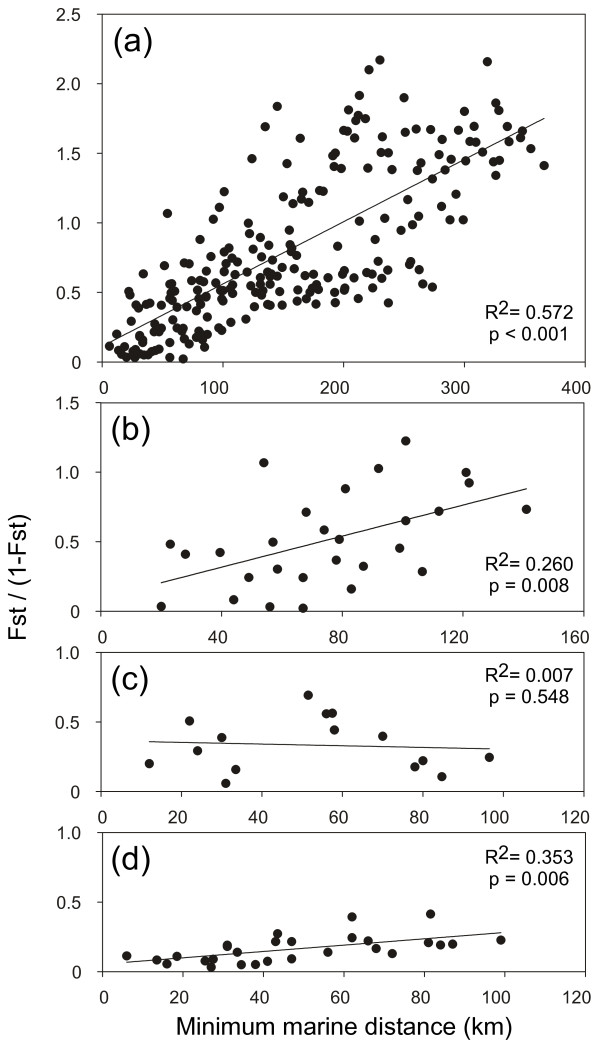
**Isolation by distance in *Fucus ceranoides *from NW Iberia. ** Estimates of pairwise differentiation (*F *_ST_/1-*F*_ST_) are plotted against geographic distance for (**a**) NW Iberia (**b**) W sector, (**c**) NW sector and (**d**) N sector. The regressions are: y = 0,0045x + 0,1134), y = 0,0056x + 0,0934, y = −0,0006x + 0,3652 and y = 0,0023x + 0,0543, respectively.

A significant IBD pattern was detected in the whole NW Iberian region (*P* = 0.001), as well as in the W (*P* = 0.009) and N (*P* = 0.009) sectors (Figure 5). In the W, however, the relationship was lost when the admixed populations of LIR and CAM were removed from the analysis (*P* = 0.135; data not shown).

## Discussion

The unprecedented spatial resolution here employed revealed a nearly perfect parapatric distribution of mtDNA lineages in the evenly distributed *Fucus ceranoides*, set by two dramatic and very narrow (<40 km) phylogeographic discontinuities (between CAM and ANL and between CED and ORT). There was a remarkable congruence between the nuclear (microsatellite) and the mitochondrial (mtIGS) data. Indeed, the multi-locus genotypes of *F. ceranoides* were broadly grouped (FCA and Structure analyses) in two main higher-level clusters, one of which was further subdivided, resulting in 3 clusters matching the mtIGS-based phylogroups.

### Allopatric divergence and secondary contact

The historical isolation, divergence and enduring integrity of three distinct and disjunct genetic pools at such a narrow spatial scale are as remarkable as they are puzzling. The congruence between the mtDNA matrilines and nuclear background shows that genetic differentiation in NW Iberian *F. ceranoides* is genome-wide, which excludes stochastic or selective sweeps as the drivers for the drastic mtDNA shifts. Also, the depth of genomic differentiation between phylogroups is high and characteristic of vicariant sub-populations that have long been diverging independently through accumulation of *de novo* mutations, drift and lineage sorting. Currently there are no large, estuarine-free regions along this shoreline and therefore distance *per se* cannot be invoked as a major contemporary factor accounting for the much deeper genomic differentiation among than within NW Iberian genetic sectors. The mean distance separating any two neighbouring populations (~32 km) and neighbouring populations across phylogeographic breaks (<40 km) are of the same order of magnitude, and several peripheral populations are geographically closer to populations across the phylogeographic breaks than they are to their nearest population within the same sector.

The parapatric divergence of Iberian *F. ceranoides* seems rather unlikely. The divergence between interior mtIGS haplotypes (*A1*, *B1* and *C1*; 4–5 mutations, K2P = 0.010–0.014) is about one fifth that between *F. ceranoides* and *F. vesiculosus* (21–23 mutations, K2P = 0.043–0.046), which are estimated to have diverged between 0.73 and 3.77 million years ago ([56], estimate based on a calibrated phylogeny of 13 nuclear genes). Assuming a constant molecular clock, the divergence of phylogroups of *F. ceranoides* could date back to 165–867 ky BP. This indirect estimate is almost certainly inaccurate, but implies that phylogroups started to diverge well before the last glacial maximum. The high levels of genetic endemism and diversity of the Iberian region agree with the expectations for long-term persistence in glacial refugia. However, the global effects of past climate and sea level changes also affected Iberian refugial areas [57-59]. The periodic transgressions and regressions associated with the expansion/melting of land-based ice-sheets caused continuous geographical rearrangement of near-shore habitats [60,61], estuaries included. The geographic locations of NW Iberian estuaries in the past were different from today, as were to some extent the climatologic, oceanographic and hydrologic regimes [58,59]. Even discounting significant changes in the density and location of NW Iberian estuaries throughout past millennia, it appears rather improbable that any oceanographic feature acting as a powerful demographic filter could have remained relatively static in approximately its current (and very narrow) positions during such a long and dynamic period.

The contemporary genetic sectors in Iberian *F. ceranoides* are more likely to result from the regional reassembly of vicariant phylogroups into their current distributions following a period of independent, mostly allopatric divergence in past contracted areas of occurrence. This scenario implies the historical fragmentation and divergence of Iberian *F. ceranoides* in separate refugia (refugia within refugia), the subsequent expansion of these vicariant phylogroups along contiguous shorelines, and very limited gene-flow across meanwhile established secondary contact zones. The mismatch analyses and the distribution of the mtIGS variation are compatible with this scenario of independent range expansions. Within each sector, the only widespread haplotypes are the interior (presumably ancestral) haplotypes, whereas derived haplotypes (presumably younger) are typically restricted to single populations. In the terrestrial realm, similar expansion/contraction cycles have been invoked to explain the mostly parapatric distribution of lineages and sister-species in a range of Iberian taxa currently displaying relatively continuous distributions (reviewed in [62-64]).

The regular climatic changes and the dynamic shoreline/drainage geography across the Pleistocene glacial/interglacial cycles [57-59] have probably played an important role in the regional range dynamics of Iberian *F. ceranoides,* but it is impossible with the present data to establish the specific drivers and its spatio-temporal contexts with detail. Our data nevertheless permit us to speculate on the temporal sequence of colonization of NW Iberia if haplotypic diversity within each sector is assumed to represent a good proxy for the time since colonization. The arrival of phylogroup C to Northern Galicia is presumed to post-date the establishment of phylogroup A in Western Iberia. Indeed, most populations of phylogroup A harbour private haplotypes in relatively high frequencies and some even local haplotype radiations. Remarkably, the number of haplotypes found in these 8 estuaries sampled along a coastline sector as small as 150 km far exceeds the number found in central and northern Europe (N_pop_ = 12; [15]). The current distribution of this phylogroup probably represents a stable interglacial rear-edge that may have experienced southward expansions during colder periods such as the last glaciation. In contrast, the entire N sector is dominated by the ancestral haplotype *C1*. Derived *C1* haplotypes were found further East, in the populations of VIL, SAN and BAY, and may indicate that phylogroup C arrived more recently to NW Iberia from an eastern Cantabrian refugium. Finally, the 6 populations forming relict phylogroup B are probably close to their refugial distribution centred on the Artabro Gulf. In the absence of complementary demographic information, however, the chronology of colonization (leading to secondary contacts) remains highly speculative.

In this contraction/expansion scenario, the enduring integrity of the fine-scale phylogeographic structure within *F. ceranoides* can only be explained by very limited gene-flow across phylogeographic breaks. Migration may be particularly depressed there due to the presence of contemporary oceanographic barriers to dispersal, or be as low as elsewhere and simply reflect the inherent low vagility of the species.

### Evidence for oceanographic barriers to dispersal is lacking

The most distinctive feature of NW Iberia coastline is its “rias”, drowned river valleys formed during the marine transgression that followed the last glaciation. These rias are generally divided, based on their orientation, size and main geomorphological elements, into “Lower Rias” (between VIG and XAL), “Middle Rias” (between LIR and CED), and “Higher Rias” (eastwards of ORT), with the transitions at Cape Fisterra and Cape Ortegal, respectively. The distribution of mtDNA lineages of *F. ceranoides* matches these subdivisions remarkably well, although establishing a link between geographic and genetic subdivisions remains difficult, as their circumscriptions are not based on distinctive climatic, hydrological or oceanographical features that could be relevant in terms of species ecology or dispersal.

The movement of buoyant, surface-drifting seaweed rafts is constrained by near-shore circulation patterns, wind, coastline morphology, tidal currents and river plumes, although the prevailing shelf/slope circulation patterns also play a role in offshore transport. It is highly challenging at the study scale to track the movements and fate of reproductive drifters leaving/arriving estuaries and therefore to directly estimate migration rates between populations within and across sectors. However, several lines of evidence suggest the absence of any specific seascape feature generating persistent (year-round) physical discontinuities matching the location of the observed genetic breaks. In NW Iberia, circulation patterns are complex and seasonally variable [65,66]. During the Autumn–Winter downwelling season, SW winds prevail and a poleward current flows over the Western slope, with inter-annual variability in intensity and penetration into the Cantabrian Sea. During the Spring–Summer upwelling season, prevailing winds shift to become predominantly NE/N oriented, and an east/southward current develops over the shelf. These characteristic patterns are intermittently dominated by short-scale meteorological events that regionally intensify or reverse circulation during short periods in each season [67]. The physical continuity of this coastline is well illustrated by the fate of the oil spilled by the Prestige tanker 250 km west off Cape Fisterra. The leaked (buoyant) fuel reached Cantabria (830 km from the sinking point) in just 17 days and spread along the Spanish shoreline from Vigo to the Basque Country [68], i.e., throughout and beyond the region studied here.

The available genetic evidence seems to confirm this. In the presence of barriers, genetic discontinuities would be expected to be concordant in location and eventually in depth, between co-occurring species sharing similar dispersal characteristics. However, most population genetic studies of shallow coastal biota focus on organisms with planktonic dispersive stages, and/or have very poor sampling resolution in the studied region. Demes from both the Western and Northern coasts of Galicia have been analysed in mussels [69], stalked barnacles [70], spider and swimming crabs [71,72], flatfish [73], octopus [74] and direct-developing cephalopods [75]. Their common characteristic is the absence of genetic structure, indicating widespread connectivity by marine currents over NW Iberia. The single exception is the low dispersal, ovoviviparous snail *Littorina saxatilis*, which showed mild subdivision north and south of Cape Fisterra [76]. The authors propose that this break coincides with a putative ecological barrier but do not exclude the alternative hypothesis that it represents a secondary contact zone where allopatrically diverged sub-populations are being homogenized very slowly. The swift spread of the invasive seaweed *Sargassum muticum* throughout Galicia, Cantabria and Portugal, shortly after its first detection in Asturias (1980) and in Galicia (1986; [77]) also fails to support oceanographic barriers to drifting seaweed dispersal in this area.

The absence of prominent seascape or ecological barriers matching the phylogeographic breaks implies two things. First, that their positions may be rather contingent and simply reflect the idiosyncratic sequence of (re)colonization of NW Iberian estuaries by the three phylogroups during past range expansions; and second, that the unusually sharp genetic discontinuities at secondary contact zones are maintained despite normal migration rates.

### Colonization history vs. ongoing gene-flow

A pattern of approximate stepping stone expansions originating in different refugia could promote the formation of genetic sectors even in the absence of dispersal barriers. This possibly reflects a relatively short viability of reproductive structures after frond dislodgement, or a density-dependent effect. *F. ceranoides* is dioecious and therefore effective estuarine colonization requires at least one male and one female fertile frond to be in close contact after dispersal (while synchronously releasing gametes) to produce *in situ* the foundational zygotes that will eventually initiate a new population. Entangled mats of drifting viable male and female *F. ceranoides* are more likely to form near established populations that may export significant amounts of freshly dislodged drifters. Successful colonization across intermediate and larger distances surely occurs, as exemplified by the haplotype sharing of ANL and CED, or the colonization of Norway (across the North Sea) and Iceland, but it is probably much less frequent. Anyway, the process will self-reinforce: if populations in the interior of a sector go extinct, favoured recolonization from nearby sources will preserve the pre-existing genetic pattern.

Rare effective inter-estuarine dispersal, while allowing the colonization of unoccupied estuaries, implies that gene-flow has little effect in counteracting differentiation between fully established populations, slowing or preventing any progress towards migration-drift equilibrium. The remarkable genetic homogeneity of northern Europe, which was colonized post-glacially, clearly demonstrates the lack of gene-flow from the interior of the species range, where *F. ceranoides* exhibits considerably more diversity [15,52]. This study goes further in demonstrating that the effects of gene-flow are remarkably insignificant even at the shortest possible scale—between consecutive estuaries. Indeed, most populations in the W and NW sectors harbour private haplotypes in relatively high frequencies, including those located inside the same drainage systems. If migration is typically so low that even consecutive populations within sectors are genetically independent, it will also be unable to readily homogenize pre-existing differentiation remaining from the colonization process.

### Density-barrier effects

The primacy of colonization history over ongoing gene-flow reflects a poorly connected metapopulation system regulated by dispersal processes that are only effective within very restricted spatial and temporal windows. The apparent paradox of extremely limited population connectivity (here reported within and between sectors) despite an evident colonization potential (at least in the long-term) suggests extreme density-barrier effects. While the initial colonizers may reproduce relatively free of competition and contribute disproportionately to the genetic make-up of establishing populations, once populations become fully established the large disparity in the number of residents (descending from the colonists) and subsequent immigrants act as a demographic buffer against changes in allele frequencies [46,51].

Organisms such as *F. ceranoides* that inhabit patchy habitats and possess the capacity for rapid population growth and habitat saturation (compared to immigration rates) may be particularly prone to density-barrier effects. *Fucus spp.* are fecund, fertilization success is typically very high and recruitment in the vicinity of parental plants can be very efficient [78,79]. Monopolization of local space by marine *Fucus* species can be fast compared to their spread along unoccupied discontinuous shores [80]. Compared to its marine congeners, *F. ceranoides* may occupy vacant estuaries even faster and to a larger extent. The enclosed and sheltered nature of its habitat should improve the number and success of spawning events [81]. Furthermore, *F. ceranoides* is a structural species that frequently forms monospecific (and often compact) belts within its particular tidal/salinity range. Contrary to the open shore where saturated communities compete for space, its estuarine habitat is free of similar competitors, potentially contributing to increased growth rates, densities and space monopolization. The sheltered and densely occupied habitat of *F. ceranoides* together with the short range of gamete dispersal of *Fucus* spp. can also explain the observed heterozygote deficiencies as reflecting local inbreeding. Other possible causes for such deviations are unlikely, as small sample sizes appear unimportant when comparing with previously analysed larger samples [52], no recurrent amplification failure (null alleles) was observed in any locus or population, and no evidence supports the co-existence of distinct but sympatric sub-populations within estuarine patches (Wahlund effects).

The limited lineage admixture reported here indeed suggests that *F. ceranoides* has relatively short temporal windows of opportunity between estuarine colonization and saturation during which rare immigration can potentially result in detectable gene-flow. Incipient reproductive isolation (pre- or post-zygotic) can add to density effects and further depress gene flow between divergent phylogroups (e.g. [41,43]). The admixed nuclear background of LIR and CAM show that these divergent lineages of *F. ceranoides* can interbreed and should not be regarded as incipient species, although hybridization is common between *Fucus* species [82,83]. Importantly, the shallow population structure within sectors shows that gene-flow is similarly reduced between more closely related populations. Unrecognized biophysical, ecological or reproductive barriers remain valid (and mutually non-exclusive) alternatives to pure demographic effects, but are probably not the most important factor underlying the apparent lack of gene-flow across sectors that maintains the parapatric structure of Iberian *F. ceranoides*.

In revealing that populations of *Fucus ceranoides* are to a large extent bounded by extinctions and (re)colonisations, our results challenge earlier assertions that marine species are mainly unified by gene-flow. In this seaweed, connectivity estimates based on allele frequency divergence (e.g. FST and related measures) are inflated (within sectors) or depressed (across secondary contact zones) to a great extent by historical colonization processes. Despite its evident parapatric structure, an IBD correlation was unexpectedly recovered in NW Iberian *F. ceranoides*, showing that IBD patterns can arise where a causal relationship between distance and gene-flow is missing. Such spurious correlations have been noted in other highly structured species [84,85], and confirm that gene-flow may not be the decisive factor underlying many significant associations between geographic and genetic distances.

## Conclusions

Our fine-scale, multi-marker approach revealed sharply disconnected population units in NW Iberian *Fucus ceranoides*. The levels of differentiation and the absence of habitat discontinuities or prominent ecological/oceanographic barriers to dispersal indicate that its remarkable genetic structure is the product of past range dynamics (including contractions, sequential expansions and secondary contact) coupled with very strong density-barrier effects. These conclusions are highly relevant to other organisms with rare and spatially restricted dispersal, helping explain the apparent paradox of extensive genetic subdivision in geographically restricted refugial regions (indicating very limited connectivity) despite obvious colonization abilities of these same species at larger spatio-temporal scales (e.g. allowing extensive post-glacial range expansions, see also [86]). These species may not fit the conventional “low-dispersal” or high-dispersal” dichotomy, since rare dispersal into vacant (colonization) and saturated (immigration) habitats can have fundamentally different demographic and genetic effects.

This study also supports the view that the patterns of genetic structure and differentiation in marine-restricted dispersers often reflect persistent non-equilibrium conditions [33]. In particular, it shows that distant (but rather similar) populations do not necessarily exchange more migrants than closer (but very divergent) populations, and that steep genetic breaks are not necessarily maintained by extrinsic dispersal barriers. The regular climatic oscillations and the transitory nature of near-shore habitats may actually prevent low dispersal marine species in general from ever attaining migration-drift equilibrium at most spatial scales. Inferring patterns of connectivity from genetic data alone may be misleading, where historical patterns of extinction and colonization are more important than ongoing gene-flow in determining the extent of genetic differentiation between extant populations.

## Methods

### Sampling, DNA isolation, sequencing and genotyping

The “core” populations of *F. ceranoides* used in this study were collected in the estuaries of all major rivers between Vigo (VIG, SW Galicia) and Navia (NAV, W Asturias), in NW Iberia (N = 22; Table 1; Figure 1). This corresponded approximately to an array of discrete but neighbouring populations with an average proximity of about 33 (±17) km. Four additional Iberian populations that are not contiguous to this “core” population set were included in some analyses; VIA (northern Portugal), VIL (eastern Asturias), SAN (Cantabria) and BAY (southern France). These and also 3 “core” populations—NOI, RCO and POR—were previously analysed by Neiva *et al.*[15,52]. All collection sites typically contained monospecific belts of *F. ceranoides* attached to hard substrata and were exposed to steep salinity fluctuations throughout the tidal cycle. At each site, 5–10 cm tips of apical vegetative tissue was excised from 16 individuals sampled along a 100–200 m linear transect or random walk; tissue samples were individually stored dehydrated in silica-gel crystals until DNA extraction. To keep sample sizes constant, a random subsample of 16 individuals was used from the previously analysed populations.

Genomic DNA was extracted from approximately 10 mg dried tissue using the Nucleospin® Multi-96 plant kit (Macherey-Nagel Duren, Germany), according to the manufacturer’s protocol. Individuals were sequenced for the mitochondrial 23 S/trnK intergenic spacer (mtIGS, [15]), and genotyped for 7 microsatellite loci developed for congeners [87-89] that had shown polymorphism in Iberian *F. ceranoides*[52]. Primer sequences and amplification details were the same as in Neiva *et al.*[15,52]. Amplified fragments were run in an ABI PRISM 3130xl automated capillary sequencer (Applied Biosystems, CCMAR Portugal). MtDNA sequences were aligned, proofread and edited in GENEIOUS 3.8 [90]. Microsatellite alleles were manually scored in STRAND [91] using the 350 ROX™ size standard (Applied Biosystems).

### Genetic structure

The geographic distribution of the mtDNA variation was mapped and the genealogic relationships of haplotypes were inferred using the median-joining algorithm implemented in Network 4.5 [92]. A phylogenetic tree for the mtDNA sequences was reconstructed with MrBayes [93] using the best-fit model of nucleotide substitution and using *Fucus vesiculosus* as the outgroup (GenBank no GQ385125). Among the 88 models evaluated in jModeltest [94,95], the HKY + G model was selected based on the Akaike information ranking. Two parallel Metropolis-coupled Markov chain Monte Carlo searches, each with four chains, were run for 2 × 10^6^ generations, sampling every 100 generations. The number of substitution rates (Nst = 2) and among-site rate variation (Rates = Gamma) were set according to the substitution model selected, leaving the remaining options as default. 10^5^ generations (1000 trees) were discarded as burn-in, and the remaining 38000 used to produce 50% majority-rule consensus trees and to calculate branch posterior probabilities.

Nucleotide (*π*_hap_) and haplotypic (*H*_hap_) diversity within populations and inferred mtIGS phylogroups (see RESULTS) were calculated with DNASP 5.10 [96]. Summary statistics of the microsatellite genetic diversity, including microsatellite allele frequencies, mean allelic richness (*A*), Nei’s gene diversity (*H*_E_), observed heterozygosity (*H*_O_) and inbreeding coefficients (*F*_IS_), were calculated with GENETIX 4.05 [97]. The partitioning of genetic variation between and among the mtIGS sectors was examined with molecular analyses of variance (AMOVA) in ARLEQUIN 3.1 [98]. The significance (*P* > 0.05) of the fixation indices was calculated after 1000 permutations of individuals within sectors. For each phylogroup, the occurrence of recent spatial expansions (assuming constant deme size) was tested with ARLEQUIN 3.1 [98], fitting the implemented model to the observed mismatch distribution. Significance was assessed with 1000 permutations.

The microsatellite population structure was assessed with both individual (genotype based) and population (allele-frequency based) approaches. First, the degree of congruence between the mtIGS structure/phylogeny and its nuclear background was visually inspected with the factorial correspondence analysis (FCA) implemented in GENETIX 4.05 [97]. Population genetic structure was further examined with a Bayesian, model-based genetic admixture analysis implemented in STRUCTURE 2.3 [99,100]. Individuals were combined into one dataset for analysis, without any *a priori* population assignments and admixture was allowed. Each number of assumed populations (*K*, set sequentially from 1 to 14) was ran five times using a burn-in of 200000 iterations and a run-length of 1000000 iterations, which was determined to be sufficient to have consistent results. The “true” number of *K* was inferred both from the posterior probability of the data, hereafter referred to as *L(K)*, and following the *ΔK* choice criterion of Evanno *et al.*[55], better suited to detect heterogeneous patterns of dispersal or co-ancestry.

Pairwise *F*_ST_ (*θ*; [101]) was estimated with GENETIX 4.05 [97] and pairwise D (*D*_est_; [102]) was estimated with SMOGD 1.25 [103]. Isolation by distance (IBD) was evaluated for full and sub data-sets using reduced major axis regressions of pairwise estimates of population’s genetic differentiation against minimum marine distance, as measured in Google Earth 5.1. The statistical significance of the genetic and geographic associations was assessed with Mantel tests (1000 randomizations, *P* < 0.05) in IBDWS [104].

## Authors’ contributions

JN conceived the study, collected samples and molecular data, performed analysis and drafted the manuscript. GAP participated in the design of the study, performed some statistical analysis and revised the manuscript. MV participated in the design of the study and revised the manuscript. EAS coordinated the study and helped draft and revise the manuscript. All authors read and approved the final manuscript.

## Supplementary Material

Additional file 1 **Figure S1.** Genetic diversity and differentiation of populations of *Fucus ceranoides* within W, NW and N sectors. **(a)** Haplotype diversity (*H*_hap_) at population (box-plots) and sector (stars) levels. **(b)** Nei’s gene diversity (*H*_E_) at population (box-plots) and sector (stars) levels. **(c)** Box-plot of pairwise differentiation of populations (*D*_est_) within regions. Box-plots depict the median (horizontal line) and the 25^th^ and 75^th^ percentiles (bottom and top of the box). Click here for file

Additional file 2 **Table S1.** Estimates of pairwise differentiation between the 26 populations of *Fucus ceranoides*. *F*_ST_ (θ) values are given above diagonal and Jost’s *D*_est_ below diagonal. Non-significant *F*_ST_ values (1000 permutations) are depicted in bold. Population codes are given in Table 1. Click here for file

Additional file 3 **Figure S2.**Most probable number of genetic clusters of Iberian *Fucus ceranoides* according to STRUCTURE. Five iterations were run for each number of genetic clusters assumed (*K*). The most probable *K* (open symbols) were inferred with Pritchard *et al.* (2000; left axis) and Evanno *et al.* (2005; right axis) choice criteria. Click here for file
