# Supplementary material for: Fine-scale genetic breaks driven by historical range dynamics and ongoing density-barrier effects in the estuarine seaweed Fucus ceranoides L
Source: BMC Evol Biol. 2012 Jun 6;12:78. doi: 10.1186/1471-2148-12-78 (PMC3483196; doi:10.1186/1471-2148-12-78)
Supplement: Additional file 1 — Figure S1. Genetic diversity and differentiation of populations of Fucus ceranoides within W, NW and N sectors. (a) Haplotype diversity (Hhap) at population (box-plots) and sector (stars) levels. (b) Nei’s gene diversity (HE) at population (box-plots) and sector (stars) levels. (c) Box-plot of pairwise differentiation of populations (Dest) within regions. Box-plots depict the median (horizontal line) and the 25th and 75th percentiles (bottom and top of the box). [file 1471-2148-12-78-S1.doc]

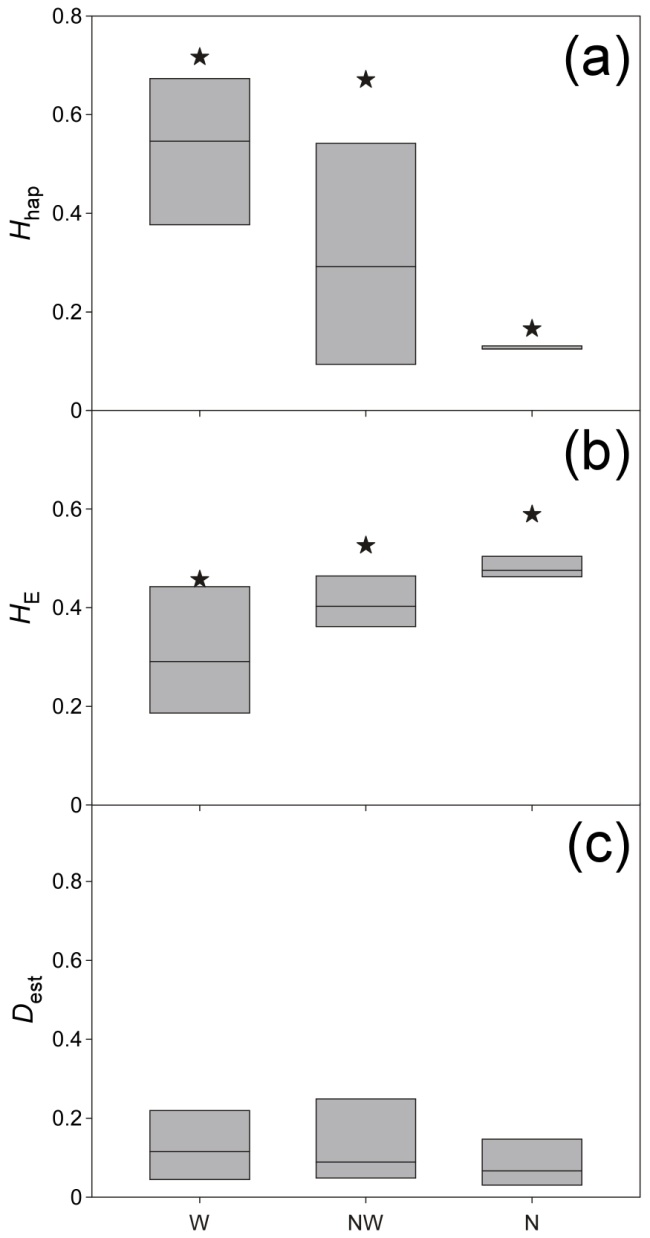


**Figure S1 - Genetic diversity and differentiation of populations of *Fucus ceranoides* within W, NW and N sectors.**

**(a)** Haplotype diversity (*H*_hap_) at population (box-plots) and sector (stars) levels. **(b)** Nei’s gene diversity (*H*_E_) at population (box-plots) and sector (stars) levels. **(c)** Box-plot of pairwise differentiation of populations (*D*_est_) within regions. Box-plots depict the median (horizontal line) and the 25^th^ and 75^th^ percentiles (bottom and top of the box).
