# Supplementary material for: Fine-scale genetic breaks driven by historical range dynamics and ongoing density-barrier effects in the estuarine seaweed Fucus ceranoides L
Source: BMC Evol Biol. 2012 Jun 6;12:78. doi: 10.1186/1471-2148-12-78 (PMC3483196; doi:10.1186/1471-2148-12-78)
Supplement: Additional file 2 — Table S1. Estimates of pairwise differentiation between the 26 populations of Fucus ceranoides. FST (θ) values are given above diagonal and Jost’s Dest below diagonal. Non-significant FST values (1000 permutations) are depicted in bold. Population codes are given in Table 1. [file 1471-2148-12-78-S2.doc]

|  | **VIA** | **VIG** | **PON** | **UMI** | **ULL** | **NOI** | **XAL** | **LIR** | **CAM** | **ANL** | **RCO** | **BET** | **ARE** | **FER** | **CED** | **ORT** | **BAR** | **VIV** | **FAZ** | **FOZ** | **VEG** | **POR** | **NAV** | **VIL** | **SAN** | **BAY** |
| --- | --- | --- | --- | --- | --- | --- | --- | --- | --- | --- | --- | --- | --- | --- | --- | --- | --- | --- | --- | --- | --- | --- | --- | --- | --- | --- |
| **VIA** | --- | 0.143 | 0.094 | 0.223 | 0.437 | 0.381 | 0.279 | 0.579 | 0.534 | 0.666 | 0.649 | 0.677 | 0.659 | 0.654 | 0.702 | 0.643 | 0.571 | 0.625 | 0.621 | 0.609 | 0.635 | 0.628 | 0.628 | 0.639 | 0.642 | 0.599 |
| **VIG** | 0.011 | --- | **0.031** | **0.021** | 0.138 | 0.312 | 0.222 | 0.499 | 0.423 | 0.617 | 0.582 | 0.618 | 0.601 | 0.601 | 0.655 | 0.598 | 0.547 | 0.591 | 0.590 | 0.592 | 0.624 | 0.605 | 0.585 | 0.607 | 0.626 | 0.576 |
| **PON** | 0.006 | <0.001 | --- | 0.076 | 0.232 | 0.340 | 0.244 | 0.550 | 0.480 | 0.648 | 0.625 | 0.657 | 0.639 | 0.636 | 0.685 | 0.626 | 0.568 | 0.615 | 0.613 | 0.612 | 0.644 | 0.629 | 0.617 | 0.631 | 0.651 | 0.601 |
| **UMI** | 0.010 | 0.001 | 0.005 | --- | **0.033** | 0.332 | 0.195 | 0.468 | 0.394 | 0.594 | 0.552 | 0.600 | 0.584 | 0.582 | 0.634 | 0.580 | 0.538 | 0.579 | 0.580 | 0.593 | 0.629 | 0.601 | 0.573 | 0.597 | 0.630 | 0.575 |
| **ULL** | 0.022 | 0.007 | 0.011 | 0.001 | --- | 0.416 | 0.269 | 0.506 | 0.418 | 0.628 | 0.597 | 0.644 | 0.624 | 0.617 | 0.677 | 0.623 | 0.589 | 0.626 | 0.625 | 0.643 | 0.683 | 0.650 | 0.613 | 0.643 | 0.683 | 0.627 |
| **NOI** | 0.071 | 0.075 | 0.075 | 0.078 | 0.097 | --- | 0.297 | 0.516 | 0.369 | 0.526 | 0.588 | 0.550 | 0.539 | 0.534 | 0.551 | 0.503 | 0.468 | 0.508 | 0.496 | 0.512 | 0.528 | 0.505 | 0.505 | 0.511 | 0.541 | 0.518 |
| **XAL** | 0.081 | 0.057 | 0.091 | 0.041 | 0.067 | 0.172 | --- | 0.291 | 0.195 | 0.414 | 0.333 | 0.388 | 0.381 | 0.381 | 0.450 | 0.377 | 0.340 | 0.377 | 0.375 | 0.398 | 0.419 | 0.399 | 0.350 | 0.393 | 0.436 | 0.381 |
| **LIR** | 0.424 | 0.368 | 0.373 | 0.297 | 0.262 | 0.596 | 0.189 | --- | 0.325 | 0.337 | 0.235 | 0.324 | 0.358 | 0.292 | 0.404 | 0.342 | 0.333 | 0.396 | 0.387 | 0.420 | 0.486 | 0.412 | 0.359 | 0.439 | 0.500 | 0.409 |
| **CAM** | 0.234 | 0.220 | 0.218 | 0.157 | 0.145 | 0.160 | 0.134 | 0.254 | --- | 0.388 | 0.431 | 0.414 | 0.393 | 0.356 | 0.393 | 0.290 | 0.304 | 0.312 | 0.299 | 0.387 | 0.391 | 0.347 | 0.298 | 0.326 | 0.473 | 0.416 |
| **ANL** | 0.937 | 0.844 | 0.879 | 0.730 | 0.673 | 0.690 | 0.607 | 0.337 | 0.516 | --- | 0.284 | 0.181 | 0.150 | 0.096 | 0.197 | 0.284 | 0.289 | 0.336 | 0.334 | 0.357 | 0.454 | 0.347 | 0.313 | 0.384 | 0.365 | 0.298 |
| **RCO** | 0.478 | 0.407 | 0.430 | 0.330 | 0.298 | 0.639 | 0.281 | 0.119 | 0.383 | 0.104 | --- | 0.226 | 0.336 | 0.279 | 0.409 | 0.369 | 0.363 | 0.442 | 0.448 | 0.472 | 0.543 | 0.457 | 0.383 | 0.505 | 0.482 | 0.430 |
| **BET** | 0.568 | 0.492 | 0.523 | 0.399 | 0.371 | 0.495 | 0.329 | 0.205 | 0.286 | 0.072 | 0.048 | --- | 0.167 | 0.136 | 0.360 | 0.318 | 0.339 | 0.427 | 0.430 | 0.456 | 0.532 | 0.434 | 0.387 | 0.482 | 0.458 | 0.365 |
| **ARE** | 0.593 | 0.507 | 0.535 | 0.416 | 0.384 | 0.536 | 0.344 | 0.301 | 0.294 | 0.076 | 0.089 | 0.024 | --- | 0.055 | 0.358 | 0.365 | 0.339 | 0.345 | 0.378 | 0.393 | 0.486 | 0.401 | 0.346 | 0.410 | 0.349 | 0.237 |
| **FER** | 0.753 | 0.655 | 0.686 | 0.557 | 0.513 | 0.646 | 0.447 | 0.227 | 0.313 | 0.048 | 0.098 | 0.064 | 0.029 | --- | 0.307 | 0.320 | 0.305 | 0.330 | 0.339 | 0.358 | 0.442 | 0.341 | 0.294 | 0.381 | 0.356 | 0.268 |
| **CED** | 0.913 | 0.814 | 0.846 | 0.706 | 0.658 | 0.556 | 0.498 | 0.307 | 0.315 | 0.112 | 0.279 | 0.249 | 0.304 | 0.250 | --- | 0.293 | 0.289 | 0.314 | 0.313 | 0.395 | 0.450 | 0.386 | 0.354 | 0.411 | 0.503 | 0.446 |
| **ORT** | 0.764 | 0.657 | 0.675 | 0.553 | 0.541 | 0.522 | 0.419 | 0.289 | 0.209 | 0.240 | 0.166 | 0.197 | 0.277 | 0.292 | 0.198 | --- | 0.031 | 0.122 | 0.122 | 0.282 | 0.293 | 0.165 | 0.185 | 0.233 | 0.368 | 0.295 |
| **BAR** | 0.698 | 0.599 | 0.594 | 0.594 | 0.672 | 0.602 | 0.434 | 0.382 | 0.331 | 0.315 | 0.240 | 0.323 | 0.305 | 0.288 | 0.289 | 0.008 | --- | 0.053 | 0.070 | 0.178 | 0.181 | 0.114 | 0.161 | 0.201 | 0.280 | 0.233 |
| **VIV** | 0.730 | 0.605 | 0.618 | 0.548 | 0.587 | 0.529 | 0.421 | 0.464 | 0.244 | 0.318 | 0.274 | 0.322 | 0.197 | 0.264 | 0.219 | 0.013 | 0.005 | --- | 0.049 | 0.214 | 0.196 | 0.143 | 0.173 | 0.160 | 0.294 | 0.261 |
| **FAZ** | 0.742 | 0.617 | 0.630 | 0.573 | 0.619 | 0.533 | 0.439 | 0.464 | 0.251 | 0.335 | 0.384 | 0.413 | 0.246 | 0.247 | 0.266 | 0.062 | 0.054 | 0.018 | --- | 0.102 | 0.082 | 0.047 | 0.085 | 0.148 | 0.346 | 0.295 |
| **FOZ** | 0.702 | 0.679 | 0.656 | 0.743 | 0.841 | 0.670 | 0.495 | 0.577 | 0.382 | 0.425 | 0.643 | 0.542 | 0.464 | 0.323 | 0.352 | 0.283 | 0.152 | 0.178 | 0.082 | --- | 0.071 | 0.153 | 0.178 | 0.197 | 0.345 | 0.286 |
| **VEG** | 0.564 | 0.594 | 0.550 | 0.662 | 0.748 | 0.513 | 0.486 | 0.587 | 0.271 | 0.605 | 0.623 | 0.638 | 0.445 | 0.374 | 0.424 | 0.247 | 0.188 | 0.151 | 0.029 | 0.026 | --- | 0.099 | 0.160 | 0.216 | 0.374 | 0.357 |
| **POR** | 0.637 | 0.551 | 0.539 | 0.568 | 0.649 | 0.490 | 0.390 | 0.383 | 0.268 | 0.407 | 0.393 | 0.450 | 0.334 | 0.304 | 0.272 | 0.101 | 0.058 | 0.072 | 0.014 | 0.095 | 0.053 | --- | 0.077 | 0.198 | 0.291 | 0.279 |
| **NAV** | 0.669 | 0.561 | 0.586 | 0.486 | 0.481 | 0.493 | 0.295 | 0.268 | 0.178 | 0.345 | 0.288 | 0.308 | 0.209 | 0.189 | 0.231 | 0.133 | 0.168 | 0.134 | 0.036 | 0.080 | 0.047 | 0.035 | --- | 0.233 | 0.317 | 0.295 |
| **VIL** | 0.756 | 0.638 | 0.666 | 0.593 | 0.639 | 0.578 | 0.437 | 0.595 | 0.291 | 0.464 | 0.559 | 0.509 | 0.400 | 0.385 | 0.356 | 0.120 | 0.164 | 0.074 | 0.059 | 0.074 | 0.082 | 0.095 | 0.106 | --- | 0.299 | 0.237 |
| **SAN** | 0.555 | 0.562 | 0.527 | 0.622 | 0.711 | 0.466 | 0.510 | 0.607 | 0.494 | 0.312 | 0.383 | 0.330 | 0.226 | 0.253 | 0.484 | 0.248 | 0.237 | 0.138 | 0.204 | 0.228 | 0.200 | 0.132 | 0.201 | 0.136 | --- | 0.136 |
| **BAY** | 0.568 | 0.509 | 0.499 | 0.537 | 0.625 | 0.626 | 0.362 | 0.444 | 0.494 | 0.338 | 0.385 | 0.339 | 0.151 | 0.185 | 0.515 | 0.271 | 0.203 | 0.225 | 0.205 | 0.210 | 0.215 | 0.115 | 0.139 | 0.139 | 0.049 | --- |

**Table S1 –** Estimates of pairwise differentiation between the 26 populations of *Fucus ceranoides*. *F*_ST_ (θ) values are given above diagonal and Jost’s *D*_est_ below diagonal. Non-significant *F*_ST_ values (1000 permutations) are depicted in bold. Population codes are given in Table 1.
