# Supplementary material for: Fine-scale genetic breaks driven by historical range dynamics and ongoing density-barrier effects in the estuarine seaweed Fucus ceranoides L
Source: BMC Evol Biol. 2012 Jun 6;12:78. doi: 10.1186/1471-2148-12-78 (PMC3483196; doi:10.1186/1471-2148-12-78)
Supplement: Additional file 3 — Figure S2.Most probable number of genetic clusters of Iberian Fucus ceranoides according to STRUCTURE. Five iterations were run for each number of genetic clusters assumed (K). The most probable K (open symbols) were inferred with Pritchard et al. (2000; left axis) and Evanno et al. (2005; right axis) choice criteria. [file 1471-2148-12-78-S3.doc]

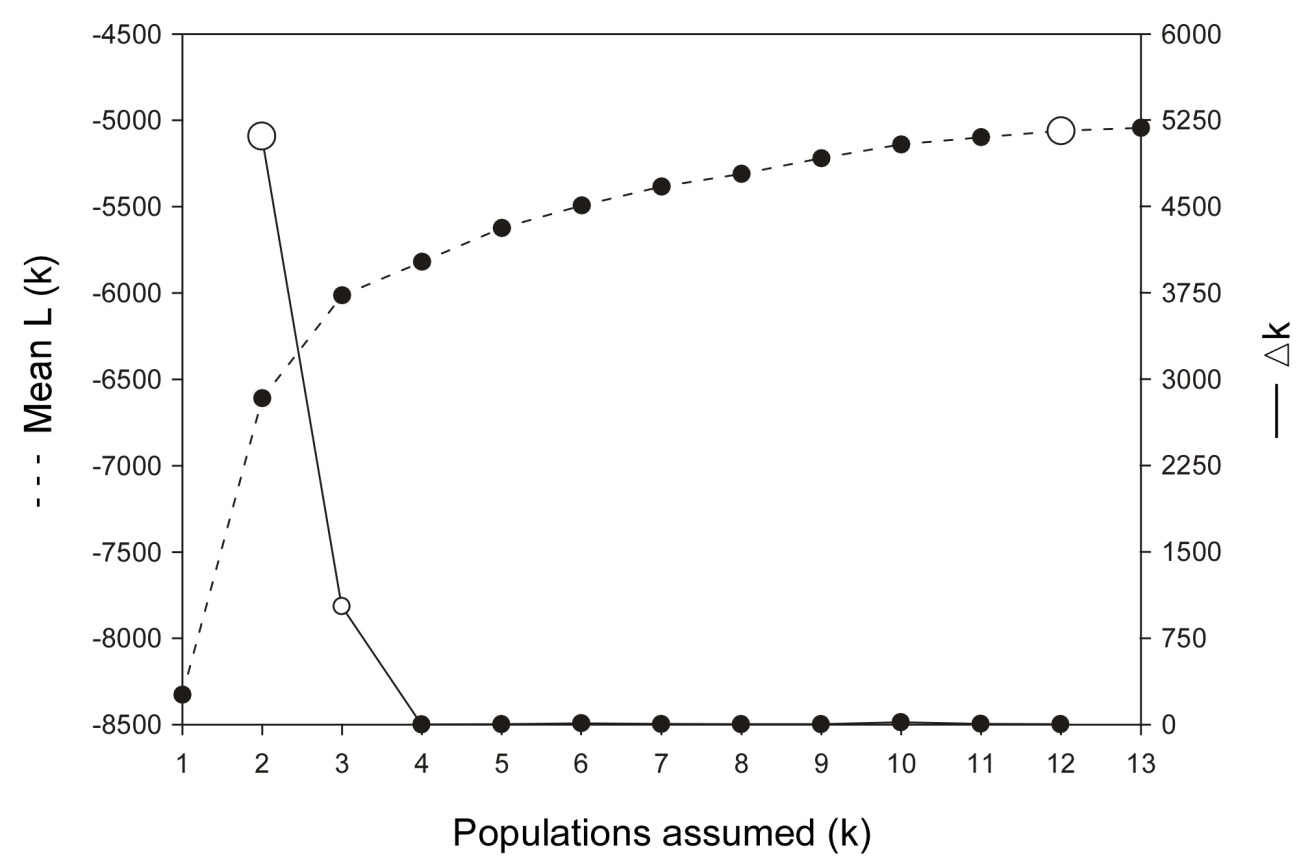


**Figure S2 – Most probable number of genetic clusters of Iberian *Fucus ceranoides* according to STRUCTURE.**

Five iterations were run for each number of genetic clusters assumed (*K*). The most probable *K* (open symbols) were inferred with Pritchard *et al.* (2000; left axis) and Evanno *et al.* (2005; right axis) choice criteria.
